# Computer-assisted drug repurposing for thymidylate kinase drug target in monkeypox virus

**DOI:** 10.3389/fcimb.2023.1159389

**Published:** 2023-05-29

**Authors:** Amar Ajmal, Arif Mahmood, Chandni Hayat, Mohammed Ageeli Hakami, Bader S. Alotaibi, Muhammad Umair, Ashraf N. Abdalla, Ping Li, Pei He, Abdul Wadood, Junjian Hu

**Affiliations:** ^1^ Department of Biochemistry, Computational Medicinal Chemistry Laboratory, Abdul Wali Khan University, Mardan, Pakistan; ^2^ Center for Medical Genetics and Hunan Key Laboratory of Medical Genetics, School of Life Sciences, Central South University, Changsha, Hunan, China; ^3^ Department of Clinical Laboratory Sciences, College of Applied Medical Sciences, Al-Quwayiyah, Shaqra University, Riyadh, Saudi Arabia; ^4^ Department of Life Sciences, School of Science, University of Management and Technology (UMT), Lahore, Pakistan; ^5^ Department of Pharmacology and Toxicology, College of Pharmacy, Umm Al-Qura University, Makkah, Saudi Arabia; ^6^ Institute of Biomedical Sciences, Shanxi University, Taiyuan, China; ^7^ Department of Obstetrics and Gynecology, Nanfang Hospital, Southern Medical University, Guangzhou, China; ^8^ Department of Central Laboratory, SSL Central Hospital of Dongguan City, Affiliated Dongguan Shilong People’s Hospital of Southern Medical University, Dongguan, China

**Keywords:** monkeypox, homology modeling, molecular docking, MD simulation, drugs development

## Abstract

**Introduction:**

Monkeypox is a zoonotic disease caused by brick-shaped enveloped monkeypox (Mpox) virus that belongs to the family of ancient viruses known as Poxviridae. Subsequently, the viruses have been reported in various countries. The virus is transmitted by respiratory droplets, skin lesions, and infected body fluids. The infected patients experience fluid-filled blisters, maculopapular rash, myalgia, and fever. Due to the lack of effective drugs or vaccines, there is a need to identify the most potent and effective drugs to reduce the spread of monkeypox. The current study aimed to use computational methods to quickly identify potentially effective drugs against the Mpox virus.

**Methods:**

In our study, the Mpox protein thymidylate kinase (A48R) was targeted because it is a unique drug target. We screened a library of 9000 FDA-approved compounds of the DrugBank database by using various in silico approaches, such as molecular docking and molecular dynamic (MD) simulation.

**Results:**

Based on docking score and interaction analysis, compounds DB12380, DB13276, DB13276, DB11740, DB14675, DB11978, DB08526, DB06573, DB15796, DB08223, DB11736, DB16250, and DB16335 were predicted as the most potent. To examine the dynamic behavior and stability of the docked complexes, three compounds—DB16335, DB15796, and DB16250 —along with the Apo state were simulated for 300ns. The results revealed that compound DB16335 revealed the best docking score (-9.57 kcal/mol) against the Mpox protein thymidylate kinase.

**Discussion:**

Additionally, during the 300 ns MD simulation period, thymidylate kinase DB16335 showed great stability. Further, *in vitro* and *in vivo* study is recommended for the final predicted compounds.

## 1 Introduction

The Mpox virus is widespread primarily in West and Central Africa and is a member of the Orthopoxvirus genus, which also include cowpox, vaccinia, variola, and smallpox (MacNeil et al., 2009). In Africa, various strains of Mpox have fatality rates ranging from 3.6% to 10.6% (Bunge et al., 2022), raising the possibility of increasing lethality. Recently, travelers have confirmed cases of monkeypox exported to Singapore (Yong et al., 2020), the United Kingdom (MacNeil et al., 2009), Israel, and the United States (Erez et al., 2019). Mpox is spread through direct contact with an animal’s body secretions or through animal bites. However, it can also be transmitted through breathing droplets during close and prolonged face-to-face contact, through direct contact with an infected person’s body fluids, or *via* objects contaminated with virus particles (Farahat et al., 2022; Lam et al., 2022).

Monkeypox is considered a sexually transmitted disease (STD) in the current outbreak. The smallpox vaccine is 85% effective at preventing monkeypox (Vallée et al., 2022). Malaise, rash, headaches, and fever between 38.5 and 40.5 degrees Celsius are some of the symptoms of monkeypox. The presence of hard, deep, and umbilicated lesions along with swollen lymph nodes are also some of the symptoms (MacNeil et al., 2009). The incubation period for Mpox lasts from one week to 17 days, with the fever going away after three days following the appearance of the rash. The lesions are described as painful, stiff, and swollen. It has been proposed that lymphadenopathy, which occurs in Mpox but not smallpox, causes a higher immune response than smallpox (Lam et al., 2022). Sepsis caused by lesions has been recorded, but it is generally believed to be uncommon (Reynolds et al., 2017). The central regions of the Mpox genome, which contain crucial enzymes and proteins, have 96.3% similarity with the smallpox genome, according to a prior study (Shchelkunov et al., 2001). The genome of Mpox is linear and has double double-stranded DNA with a genome size of 197 kb (Kugelman et al., 2014). A48R is a thymidylate kinase that has previously been found to bind with thymidine diphosphate. It is a unique target because no recognized drugs currently target it. The substantial structural difference from human thymidylate kinase at the active site makes it an attractive target (Caillat et al., 2008). According to reports, smallpox immunizations are 85% effective against monkeypox (Fine et al., 1988) (Grant et al., 2020) (Reynolds and Damon, 2012),,. Since the illness was eradicated in 1980, smallpox immunization programs have ceased (Belongia and Naleway, 2003). Although the use of smallpox medications against monkeypox is advised (Rizk et al., 2022), their safety and efficacy in human beings have not yet been determined (Sherwat et al., 2022). The present surge, therefore, indicates the urgent need for developing Mpox-specific drugs and therapies.

Finding new drugs requires several steps, including target identification, lead identification, animal studies, and clinical trials, which can typically take at least ten to twelve years to complete. Consequently, drug repurposing is a desirable substitute in the epidemic’s situation (Yong et al., 2020). The strategy of repurposing drugs has numerous advantages, including a significant decrease in testing time (Sherwat et al., 2022). In particular, drugs that have already received approval for treating different illnesses have undergone comprehensive toxicity testing and can thus be given safely to the general public (Yong et al., 2020). The drug discovery process now includes computational methods that allow the screening of small molecule libraries to identify lead candidates that can be optimized to find potential medications for clinical testing (Ashburn and Thor, 2004) (Sliwoski et al., 2014),. The crystal structure of the TMPK enzyme of Mpox was not available in the PDB database so we developed a homology model of the TMPK drug target. Furthermore, a total of 9000 FDA-approved drugs retrieved from the drug bank database were screened against the drug target. In order to computationally evaluate the stability of the ligand-protein complexes, we then carried out molecular dynamics (MD) simulations for the top three inhibitors predicted by the docking studies. In summary, our *in-silico* research explores whether drug that have already been approved can be good candidates against Mpox.

## 2 Materials and method

### 2.1 Structure prediction

In computational biology, predicting protein structure has been a significant scientific challenge for a long time. Regardless of the size of a protein, homology modeling has proved a relatively quick method for predicting a protein’s structure based on experimental structures available in the Protein Data Bank. AlphaFold is a trained neural network-based deep learning method for homology modeling. The most recent version of this software, AlphaFold 2, revealed extremely high accuracy in predicting protein structures (Cramer, 2021). In the present study, Google Colab was used for the prediction of the 3D structure of thymidylate kinase (TK). For the purpose of evaluating Ramachandran plots to confirm the stereochemical accuracy of the predicted protein structure, PROCHECK (Suganya et al., 2014) was used. ERRAT (Zheng et al., 2022) was also used for model validation.

### 2.2 Structure-based virtual screening and docking

#### 2.2.1 Structure preparation

It is crucial to employ precise protein structures in structure-based molecular modeling. In this study, the model developed by AlphaFold 2 was used for virtual screening. The structure preparation wizard of MOE (Molecular Operating Environment 2016) software was used to prepare the structure (Ahmed et al., 2022). The protein structure was 3D protonated to add hydrogen atoms, and then the MOE software default parameters were utilized to minimize the energy of the structure.

#### 2.2.2 Ligands preparation

Over 9000 compounds documented in the drug-bank database were retrieved from the drug-bank database. All of the compounds in the drug-bank database were subjected to three-dimensional protonation using the MMFF94x force field and energy minimization using MOE software with an RMS gradient of 0.05 (Rauf et al., 2022).

#### 2.2.3 Molecular docking

Structure-based virtual screening (SBVS) techniques require the 3D structures of the drug target (receptor) and ligands in the database. We used molecular docking to evaluate the binding patterns of drug-target proteins and ligands in order to discover novel potential inhibitors. The interactions between ligands and receptors can be predicted by molecular docking approaches. The molecular docking investigations were carried out using the MOE docking program (Taj et al., 2022). The retrieved compounds were docked with the active site of thymidylate kinase to predict the active compounds against thymidylate kinase (A48R). For each compound, a total of five conformers were generated, and the top-ranked conformation for each compound was analyzed for interaction analysis. Using the rigid receptor docking protocol and GBVI/WSA scoring function, molecular docking (Taha et al., 2023) of all FDA-approved drugs was performed. AutoDock Vina software was used to carry out the docking operation (Ali et al., 2023) of the lowest docking score hits obtained from the MOE software to validate the docking protocol of the MOE software. Adding polar hydrogen atoms and assigning partial charges to each atom were all done using the AutoDock software, and the structure of ligands and receptors were saved in PDBQT format. The grid spacing was set to 1 Å and the box size was 16×20 ×24. Twenty conformations were set in the docking output. The binding poses of the molecule with the best docking energies were visualized using the PyMol software (Du et al., 2023).

### 2.3 MD simulation

Molecular dynamics simulations were performed using the AMBER 20 software suite (Ajmal et al., 2022). The best docking score complexes were used as the starting structures for the MD simulations. The AMBER force field FF14SB was used for the protein, and the generic AMBER force field (GAFF) was used for the ligands (Wadood et al., 2022). The topology files and atomic charges of ligands were generated using the Antechamber suite in the AMBER 20 package (Mahmood et al., 2023). The topology and coordinate data for the entire system were generated using the tleap module of the AMBER 20 software. The entire system was soaked in a TIP3P water box at a margin distance of 8 Å. An appropriate quantity of chloride ions was introduced in order to neutralize the system’s charge. During the simulation, the particle mesh Ewald (PME) was used to handle the long-range electrostatic interactions, and the cut-off distance for nonbonded interactions was adjusted to 10 Å. The SHAKE algorithm (Poli et al., 2022) was employed to constrain the hydrogen-containing bonds. Each system underwent two stages of energy minimization: the first stage involved the use of the algorithms (10,000 steps of the steepest descent and 10,000 steps of the conjugate gradient) with restraint, and the second stage involved the use of the same algorithms without restraint. Then, each system was gradually heated from 0 to 300 K. The system was then brought up to equilibrium at 300 K and constant pressure. Finally, a production process lasting 200 ns was carried out under conditions of constant temperature and pressure (NTP). Finally, the CPPTRAJ was used for trajectory analysis (Junaid et al., 2018).

#### 2.3.1 Principal component analysis

Proteins’ high-amplitude movements can be captured by using principal component analysis (PCA). The PCA analysis in this study was performed using the cpptraj package (Ajmal et al., 2022). The covariance matrix was generated based on the Cartesian coordinates of the carbon alpha of each system to evaluate the dynamic behavior of each system. The covariance matrix’s eigenvalues and eigenvectors were obtained by diagonalization. The eigenvectors and eigenvalues, respectively, showed the direction of high-amplitude motion and their mean square fluctuation. The PC1 and PC2, for each system, were calculated and plotted to track their movements.

### 2.4 Binding energy calculation

The free energy of binding between ligands and protein complexes was computed by the MMGBSA and MMPBSA techniques. For the calculation of binding energy, the last 100 frames were used. Two effective methods for analyzing the free energy of binding are MM/GBSA and MM/PBSA. The values of MM/PBSA strongly correlate with experimental methods (33). Here, we calculated the binding free energy using both the MMPBSA and MMGBSA method. The following equation was employed to calculate the free energy:


Gbind=ΔGcomplex−[ΔGreceptor+ΔGligand]

In the above equation, *Gbind* stands for the total binding free energies, *ΔGcomplex* for complex free energies, and the remaining terms stand for the corresponding free energies of the receptor protein and ligand.

## 3 Results

### 3.1 Protein sequence retrieval

The protein sequence of the Mpox virus thymidylate kinase was retrieved from the NCBI database.

### 3.2 Homology modeling

**Figure 1:**
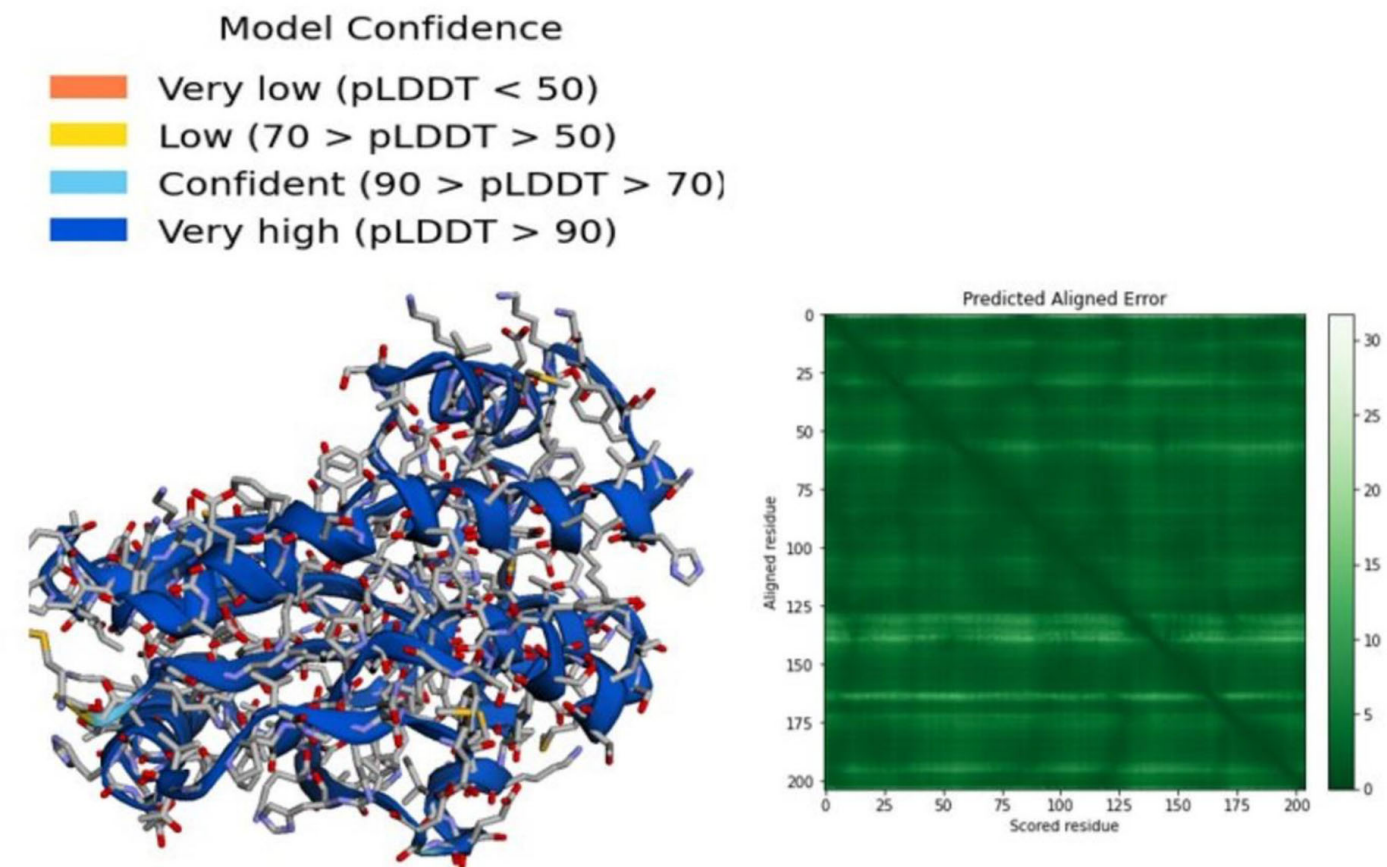
3D structure of thymidylate kinase, a drug target in Mpox, generated by AlphaFold 2 server.

In addition, the 3D structure was predicated by the AlphaFold2 server as presented in **Figure 1**. A per-residue confidence rating between 0 and 100 was produced by AlphaFold2. There may be some disorder regions in the predicted structure which can be represented by a low pLDDT value. In the generated model, most of the residues have very high confidence scores that represent the model accuracy (pLDDT > 90, **Figure 1**). The dark green region in the predicted aligned error plot is ideal, indicating the accuracy of the model, but light green is bad (indicating a high error).

### 3.3 Structure validation

The structure was validated by PROCHECK and ERRAT servers. PROCHECK is used for three-dimensional model validation. The Ramachandran plot is shown in **Figure S1A**. In the plot, residues were determined in three various regions such as favored region (94%), allowed region (4.3%), and outlier region (0.5%). Furthermore, the ERRAT server was used to check the quality of the model. ERRAT validated the model by using statistical relation of non-bonded interactions between various atom types on basis of atomic interaction. The values around 95% and higher than 95% indicate standard high-resolution structures; however, values around 91% indicate low-resolution structures. **Figure S2B** illustrates a 97% ERRAT score, indicating the best model according to the ERRAT plot.

### 3.4 Molecular docking analysis

**Figure 2:**
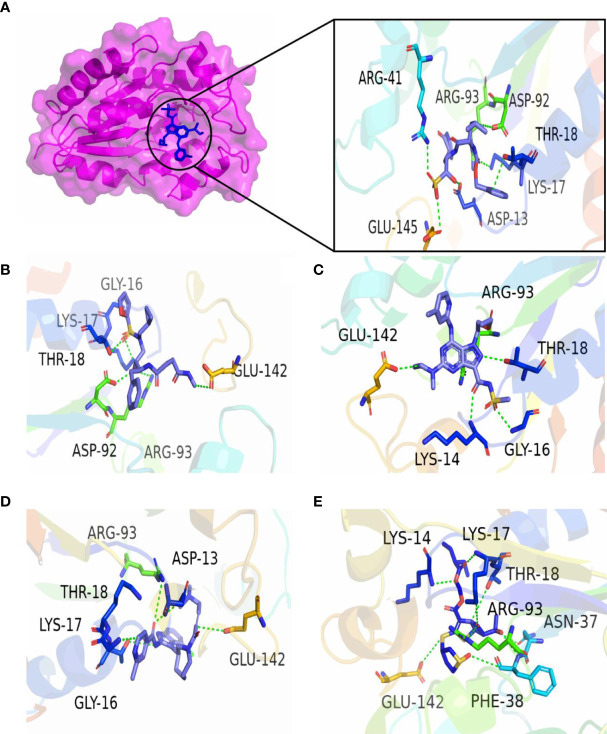
3D interactions of top five final compounds: **(A)** DB15796; **(B)** DB08223; **(C)** DB11736; **(D)** DB16250; and **(E)** DB16335 in complex with thymidylate kinase. The green dotted line represents the bond.

In the present study, molecular docking was performed to evaluate the interaction of FDA-approved drugs against the drug target thymidylate kinase. By employing the site finder option of MOE software, active site residues were identified. The docking scores of all the compounds were arranged in ascending order. Twenty percent of the data was further used for interaction analysis. Among these compounds, five compounds exhibited the most active compounds against thymidylate kinase. As noted in **Figure 2A**, compound DB15796 mediates a docking score of -7.49 kcal/mol. Compound DB15796 made six hydrogen bonds with the active site residues of thymidylate kinase (Asp13, Asp92, Glu145, Lys17, Arg41, Arg93), and one π-H (Thr18) interaction (**Table 1**). Moreover, in **Figure 2B**, the compound DB08223 exhibited six hydrogen bonds with Lys17, Gly16, Thr18, and Arg93 with a docking score of -7.12 kcal/mol. The docking complex of DB11736 and A48R exhibits hydrogen bonds with Thr18, Lys14, and Gly16, one ionic (Glu142), and one Pi-cation (Arg93) interaction with a docking score of -7.29 kcal/mol as depicted in **Figure 2C**. In addition, the complex of DB16250 and A48R protein, as shown in **Figure 2D**, mediates one hydrogen bond donor (Asp13), three hydrogen bonds acceptor (Gly16, Lys17, Arg93), one ionic bond (Glu142), and one π-cation (Thr18) interactions. The docking score of the compound DB16250 was predicted as -8.65 kcal/mol. Finally, the compound DB16335 exhibits the best docking score of -9.57 kcal/mol and made seven hydrogen bonds with Lys 14, lys17, Thr 18, Asn37, Arg93, Phe38, and Glu142 residues of thymidylate kinase (**Figure 2E**) (**Table 1**). A number of studies reported that the success rate of virtual screening can be increased by combining the results of different docking software (36). To increase the success rate of virtual screening, the top best docking score hits obtained from the MOE software were further docked by the AutoDock software. The binding energy values obtained from AutoDock Vina are presented in **Table 2**. The binding energy obtained from AutoDock and the S score obtained from the MOE software is almost similar and ranges from -7.5 to -9.4 kcal/mol. The docking results also revealed that by performing the docking with MOE and AutoDock software, the residues including Asp13, Lys17, Thr18, Arg93, Arg41, and Gly16 were involved in the hydrogen bond formations with the ligands atoms.

**Table 1:** Docking score and protein-ligands interaction of best-scored compounds.

Compound ID	Docking score (S)	Receptors			Interactions	Distance	E(kcal/mole)
DB15796	-7.49	OE1 OD2 OD1 NH2 NZ NE N	GLU ASP ASP ARG LYS ARG THR	145 92 13 41 17 93 18	H-bond H-bond H-bond H-bond H-bond H-bond π-H	3.14 3.20 2.77 2.86 2.87 3.01 4.35	-1.3 -0.7 -0.8 -4.8 -11.8 -2.0 -0.6
DB08223	-7.12	OD2 OE2 NE NZ N N	ASP GLU ARG LYS THR GLY	92 142 93 17 18 16	H-bond H-bond H-bond H-bond H-bond H-bond	2.94 2.95 2.20 2.90 2.84 3.23	-3.5 -1.4 98.8 -5.8 -3.8 -1.5
DB11736	-7.29	OG1 N N OE2 NE	THR LYS GLY GLU ARG	18 14 16 142 93	H-bond H-bond H-bond Ionic π-cation	2.89 3.03 2.73 3.33 3.93	-3.6 -3.8 -3.3 -2.6 -2.1
DB16250	-8.65	OD2 NH2 NZ N OE2 N	ASP ARG LYS GLY GLU THR	13 93 17 16 142 18	H-bond H-bond H-bond H-bond Ionic π-H	3.51 2.73 3.40 2.93 3.92 4.10	-1.0 -5.7 -1.6 -1.0 -0.7 -1.6
DB16335	-9.57	O OE2 CB NZ NE N N	PHE GLU ASN LYS ARG LYS THR	38 142 37 17 93 14 18	H-bond H-bond H-bond H-bond H-bond H-bond H-bond	2.84 2.57 2.64 3.50 2.96 3.22 3.26	-1.6 0.5 1.2 -2.4 -1.0 -3.0 -1.3

**Table 2:** The binding energy and interacted residues of the docked compounds obtained from the AutoDock vina software.

Drug bank ID	Binding energy	Interacting residues
DB15796	-8.1	ASP 13, LYS 17, THR 18, ARG 93
DB08223	-8.4	ASP 13, GLU 145, PHE 38, LYS 14, ARG 41, ARG 93
DB11736	-7.6	THR 18, THR 19, ASN 37, ARG 93
DB16250	-7.5	PRO 39, LYS 14
DB16335	-9.4	ASP 13, THR 18, ARG 41, GLY 16

### 3.5 Molecular dynamic simulation analysis

Molecular dynamic simulation helps to study conformational changes of compounds (ligands) into the binding pocket of a protein. On the basis of docking score and interactions, the top three compounds—DB16335, DB15796, DB16250—and Apo state of thymidylate kinase were subjected to MD simulation in which dynamic behavior and stability of complexes were examined by utilizing Amber 20 software. Further, RMSD values were analyzed to evaluate the stability of complexes.

#### 3.5.1 Stability analysis

**Figure 3:**
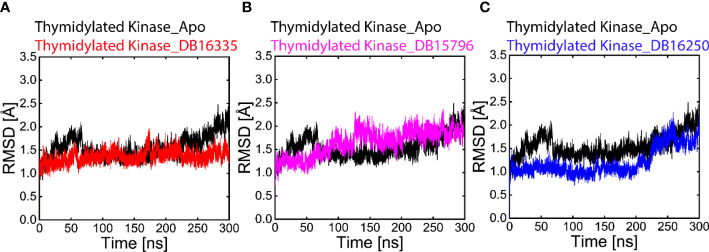
Black-colored lines in all graphs represent the Apo state. **(A)** Root-mean-square deviation (RMSD) graph of thymidylate kinase DB16335, **(B)** thymidylate kinaseDB15796, **(C)** graph of thymidylate kinase DB16250.

In the Apo state, initially, RMSD was drastically high at 2.0 Å up to 70 ns. After that, the RMSD value decreases to 1.4 Å from 80 to 230 ns. The RMSD value then gradually increases to 2.2 Å up to 300 ns. The average RMSD of the Apo-state was found to be 2.2 Å. The thymidine kinase DB16335 compound has a minimum RMSD value of 1.2-1.5 Å up to 160 ns, representing maximum stability in trajectories, however during 160 to 170 ns, small fluctuations were observed, and then after 180 to 300 ns, the complex revealed great stability and remained stable throughout the 300 ns (**Figure 3A**). The thymidine kinase DB15796 initially showed stable behavior with an RMSD value of 1.2 Å up to 70 ns, then the RMSD value gradually increases to 2.2 Å up to 80-250ns, and finally, becomes stable at 300 ns (**Figure 3B**). The thymidylate kinase DB16250 shows a steady state behavior with an RMSD value of ∼1.5 Å at 25 ns up to 230 ns. After that, the RMSD increases to 2.4 Å up to 270 ns and then reaches a stable condition at 300 ns (**Figure 3C**). Among all the complexes, the RMSD of the thymidine kinase DB16335 complex was more stable.

#### 3.5.2 Residues flexibility index analysis

**Figure 4:**
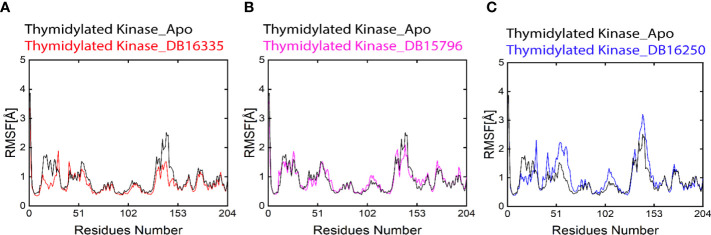
Black-colored lines in all graphs represent the Apo state. **(A)** Root-mean-square deviation (RMSD) graph of thymidylate kinase DB16335; **(B)** thymidylate kinase DB15796; **(C)** thymidylate kinase DB16250.

RMSF analysis was performed in order to evaluate the contributions of every amino acid to the stability of complexes. The residues from 50-53 and -150-153 show major fluctuations in all complexes during simulation (**Figure 4**). Smaller fluctuations represent a stable complex. The overall result of RMSF indicates that thymidine kinase DB16335 and thymidine kinase DB15796 show low fluctuations compared with Apo; however, thymidylate kinase DB16250 shows more similar fluctuations to that of the Apo state. Residues Asp 50, Asp51, Tyr 52, Leu 53, Gln 151, Lys 152, and Val 153 revealed high fluctuations during the 300 ns MD simulation. The RMSF plot is shown in **Figure 4**.

#### 3.5.3 Compactness analysis

**Figure 5:**
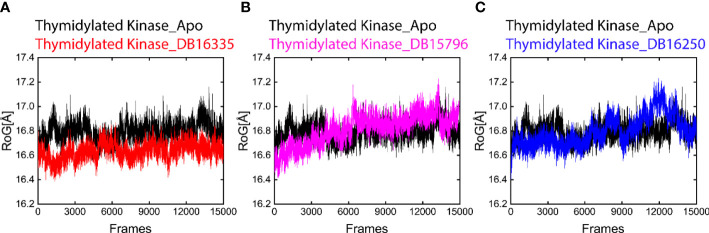
Radius of gyration (RoG) plot of Apo (black) in all graphs. **(A)** ROG graph of thymidylate kinase DB16335; **(B)** thymidylate kinase DB15796; **(C)** thymidylate kinase DB16250.

Folded and unfolded states of protein complexes were predicated by RoG. Our result revealed that the final three complexes along with the Apo state represent change behaviors throughout molecular dynamic simulations. In addition, we also analyze the radius of gyration (RoG) that helps to measure the compactness of the complexes. The average ROG values of thymidine kinase DB16335, thymidine kinase DB15796; thymidylate kinase DB16250; and Apo were 16.5 ± 16.7; 16.5 ± 16.9; 16.6 ± 17.0; and 16.7± 17.1 A, respectively (**Figure 5**). Among all the complexes as well as the Apo state, the complex thymidine kinase DB16335 revealed a highly compact complex.

#### 3.5.4 Principal component analysis

A dimensionality reduction technique such as principal component analysis was performed to examine the mobility of the proteins and the clusters of the relevant structural frames.

**Figure 6:**
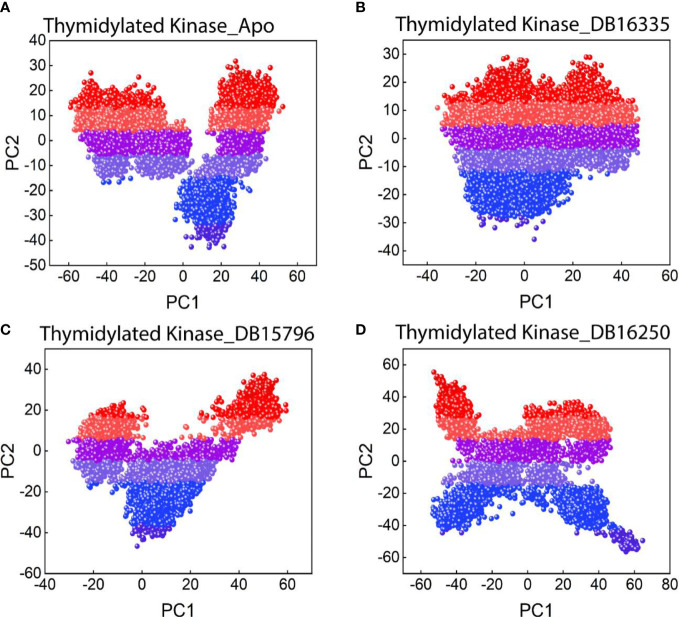
PCA plot for **(A)** thymidylate kinase-Apo state; **(B)** thymidylate kinase DB16335; **(C)** thymidylate kinase DB15796; **(D)** thymidylate kinase DB16250.

A statistical technique known as principal component analysis (PCA) combines several correlated variables with a smaller set of uncorrelated variables called principal components. The PCA was calculated and is displayed in **Figure 6** in order to properly evaluate the effect of the drug binding on protein mobility. The first two principal components, known as PC1 and PC2, were plotted against one another. Colors ranging from red to blue reflect how one conformation changes into another. Each dot in **Figure 6** corresponds to a single frame of the trajectory. The coordinate covariance matrix, which was derived from the time series of 3D positional coordinates of the complexes over the course of the 200 ns MD simulation, served as the input for principal component analysis. The Apo state displayed a slightly dispersed form of motion, while the drug complexes displayed a cluster type of motion. Among all the complexes TK DB16335 revealed more cluster types of motion.

### 3.6 Binding energy calculations

Finally, we calculated ΔG binding in order to refine ligands predicted through virtual screening by using MM/PBSA and MM/GBSA techniques. The MMGBSA analysis composed of electrostatic energy, van der Waals energy, surface area energy, and electrostatic contribution to the solvation-free energy was calculated as presented in **Table 3**, while **Table 4** represents the MMPBSA analysis. Among all the complexes, DB16335 in complex with thymidylate kinase revealed lower binding energy and high binding affinity for thymidylate kinase, a drug target in the Mpox virus predicted by both MMPBSA and MMGBSA methods.

**Table 3:** MMGBSA analysis reported in kcal/mol.

Complexes	^VDW^	^EEL^	^ESURF^	^EGB^	ΔG ^Total^
DB16335	-63.6066	-3.5111	-6.7332	13.7796	-60.0723
DB15796	-27.2213	-19.2840	-2.9749	29.3219	-20.1555
DB16250	-46.2392	0.5465	-5.4155	15.6348	-35.4764

**Table 4:** MMPBSA analysis reported in kcal/mol.

Complexes	^VDW^	^EEL^	^ENPOLAR^	^EDISPER^	^EPB^	ΔG ^Total^
DB16335	-63.6066	-3.5111	-33.8273	65.3356	20.5451	-15.0652
DB15796	-27.2213	-19.2840	-15.7229	30.8764	31.6112	0.2622
DB16250	-46.2392	0.5465	-26.2276	53.4729	24.8560	6.4056

## 4 Discussion

Mpox virus, a member of Orthopoxvirus genus that includes smallpox, has become endemic to Africa and throughout the world. No vaccines against Orthopoxviruses have been developed in the last four decades, after the eradication of smallpox. The reemergence of monkeypox in unprepared and unvaccinated populations is an emergency demanding attention from the scientific community. Instead of infeasible mass vaccination campaigns in a short time period, specific therapeutics against Mpox can provide sustainable solutions for affected patients (Potter et al., 2007). Approximately 49 genes are common among all the members of the provirus family from the total 150 genes encoded by poxviruses (Llanos et al., 2021). Currently, no approved drug is available for the treatment of monkeypox virus (Anwar et al., 2023).

Drug repurposing is best way to determine effective therapeutics against Mpox. Therefore, the present study aims to identify the best drugs against Mpox by using different computational approaches such as homology modeling, virtual screening, molecular docking, and MD simulation. For this purpose, we collected FDA-approved drugs, and 3D structure of protein was modeled. The model structure was subjected to molecular docking against a curated library of FDA-approved drugs. Afterwards, by identifying binding energy and interaction, various compounds were selected in which drugs DB12380, DB13276, DB13276, DB11740, DB14675, DB11978, DB08526, DB06573, DB15796, DB08223, DB11736, DB16250, and DB16335 mediate significant binding energy and binding interaction with thymidylate kinase (A48R). Among these compounds, the compounds having the lowest binding energy—DB15796, DB08223, DB11736, DB16250, and DB16335, with -7.49, -7.12, -7.29, 8.65, and -9.57 kcal/mol, respectively—were recognized as the best compounds (**Table 1**).

Finally, compounds DB16335, DB15796, and DB16250 along with Apo were subjected to 300 ns MD simulation to evaluate the stability of the compounds. Among the Apo and selected compounds, DB16335 was found to be active and showed strong binding against Mpox. DB16335 is an antibiotic drug that is under a clinical trial NCT03354598) and is used to treat complicated and uncomplicated urinary tract infections, intra-abdominal infections, pneumonia, and acute pyelonephritis. In addition, it is also active against Gram-positive and Gram-negative bacteria (Dunne et al., 2023). The selected compounds have low RMSD and low amino acid residue fluctuations compared with Apo, which indicates that the binding of selected compounds to the active site of Mpox protein makes the protein more stabilized. In addition, the ROG also indicates that thymidylate kinase DB16335 has a low value of RoG (**Figure 5**). The binding of these compounds to Mpox can destroy its activity and provide an unfavorable situation for Mpox.

## 5 Concluding remarks

Monkeypox has rapidly spread through the world, but still, no suitable vaccine or drug is available. The present study is designed to determine a potent drug against Mpox by using computational methods. Our result shows that all three compounds were notable because of good interaction and binding energy. Among all the drugs, DB16335 was identified as the most potent compound in terms of docking score, interactions, and binding energy calculation. Overall, compound DB16335 could bind and inhibit the drug target thymidylate kinase, which can be helpful to reduce infections associated with the Mpox virus. However, further *in vitro* and *in vivo* experiments are required to validate the findings of our study.

## Data availability statement

The raw data supporting the conclusions of this article will be made available by the authors, without undue reservation.

## Author contributions

AA, AM and CH performed experiments, drafted the manuscript. MAH performed some of the experiments and revised the manuscript and discussion. BSA, PL, MU, ANA and PH drafted the manuscript and critically revised the manuscript. AW and JH conceptualized, designed, and supervised the study and revised the manuscript. All authors contributed to the article and approved the submitted version.
