# Supplementary material for: Computer-assisted drug repurposing for thymidylate kinase drug target in monkeypox virus
Source: Front Cell Infect Microbiol. 2023 May 29;13:1159389. doi: 10.3389/fcimb.2023.1159389 (PMC10258308; doi:10.3389/fcimb.2023.1159389)
Supplement: Supplementary file 1 [file DataSheet_1.docx]

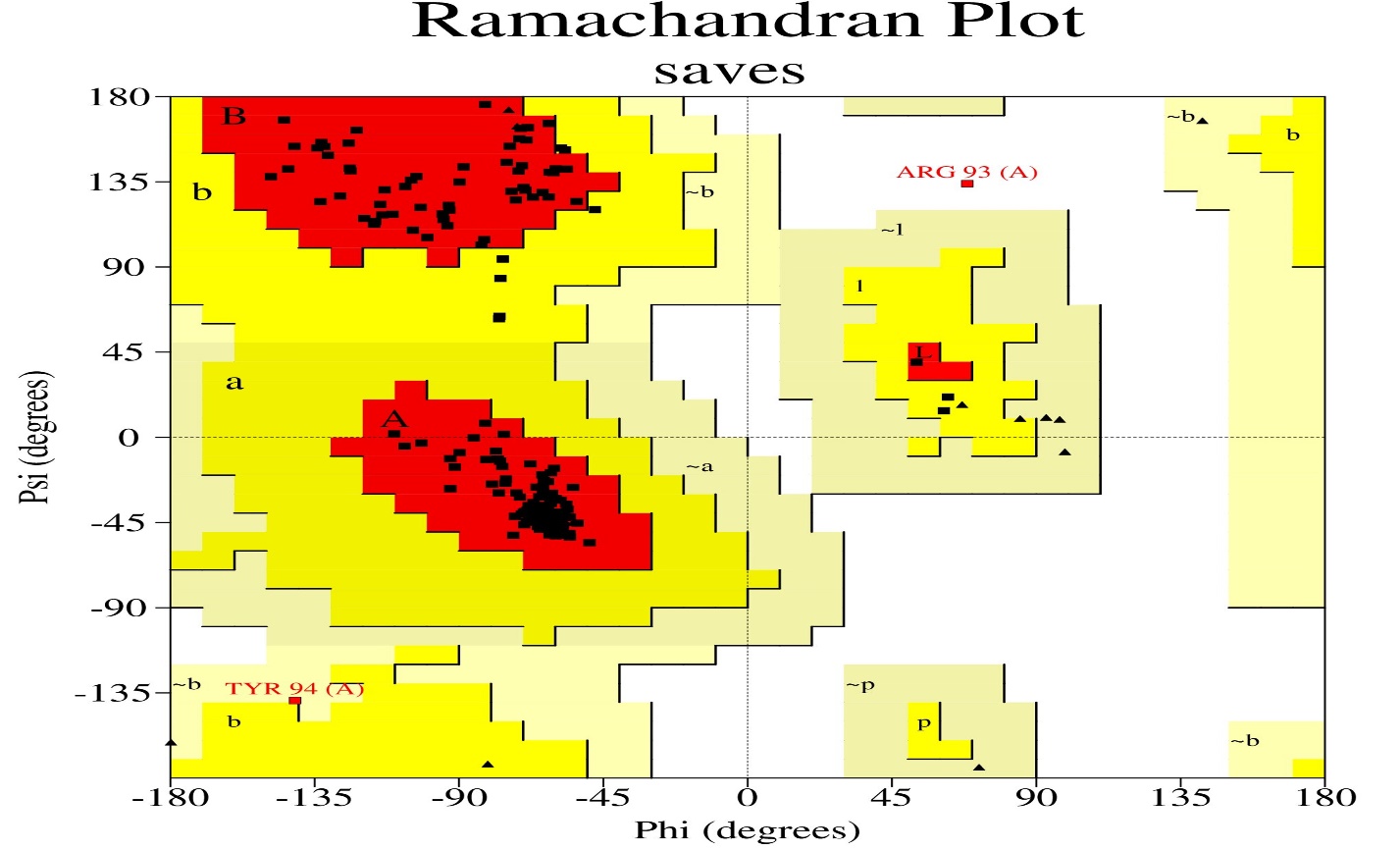


**Figure S1A.** Ramachandran plot of the developed model of thymidylate kinase.


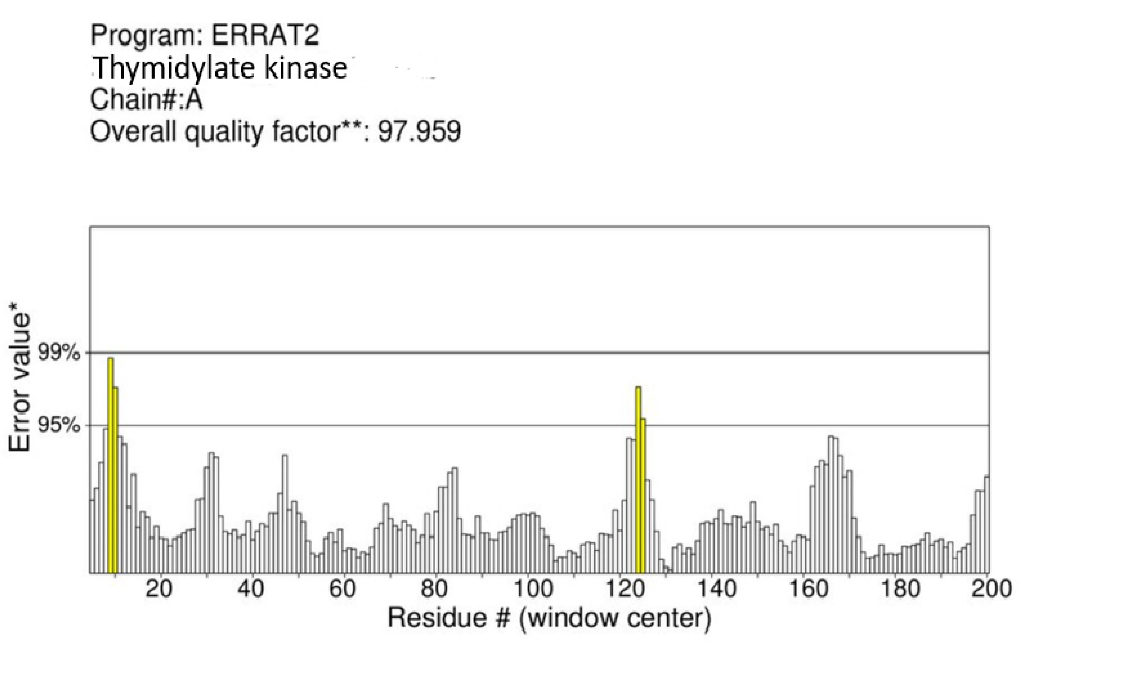


**Figure S1B.** ERRAT plot of the developed model of thymidylate kinase.
